# Type III Interferon-Mediated Signaling Is Critical for Controlling Live Attenuated Yellow Fever Virus Infection *In Vivo*

**DOI:** 10.1128/mBio.00819-17

**Published:** 2017-08-15

**Authors:** Florian Douam, Yentli E. Soto Albrecht, Gabriela Hrebikova, Evita Sadimin, Christian Davidson, Sergei V. Kotenko, Alexander Ploss

**Affiliations:** a Department of Molecular Biology, Princeton University, Princeton, New Jersey, USA; b Department of Pathology, Rutgers Cancer Institute of New Jersey, Rutgers Robert Wood Johnson Medical School, New Brunswick, New Jersey, USA; c Department of Microbiology, Biochemistry and Molecular Genetics, Center for Immunity and Inflammation, Cancer Institute of New Jersey, New Jersey Medical School, Rutgers Biomedical and Health Sciences, Newark, New Jersey, USA; GSK Vaccines

**Keywords:** flavivirus, innate immunity, interferons, live vector vaccines, yellow fever virus

## Abstract

Yellow fever virus (YFV) is an arthropod-borne flavivirus, infecting ~200,000 people worldwide annually and causing about 30,000 deaths. The live attenuated vaccine strain, YFV-17D, has significantly contributed in controlling the global burden of yellow fever worldwide. However, the viral and host contributions to YFV-17D attenuation remain elusive. Type I interferon (IFN-α/β) signaling and type II interferon (IFN-γ) signaling have been shown to be mutually supportive in controlling YFV-17D infection despite distinct mechanisms of action in viral infection. However, it remains unclear how type III IFN (IFN-λ) integrates into this antiviral system. Here, we report that while wild-type (WT) and IFN-λ receptor knockout (λR^−/−^) mice were largely resistant to YFV-17D, deficiency in type I IFN signaling resulted in robust infection. Although IFN-α/β receptor knockout (α/βR^−/−^) mice survived the infection, mice with combined deficiencies in both type I signaling and type III IFN signaling were hypersusceptible to YFV-17D and succumbed to the infection. Mortality was associated with viral neuroinvasion and increased permeability of the blood-brain barrier (BBB). α/βR^−/−^ λR^−/−^ mice also exhibited distinct changes in the frequencies of multiple immune cell lineages, impaired T-cell activation, and severe perturbation of the proinflammatory cytokine balance. Taken together, our data highlight that type III IFN has critical immunomodulatory and neuroprotective functions that prevent viral neuroinvasion during active YFV-17D replication. Type III IFN thus likely represents a safeguard mechanism crucial for controlling YFV-17D infection and contributing to shaping vaccine immunogenicity.

## IMPORTANCE

YFV-17D is a live attenuated flavivirus vaccine strain recognized as one of the most effective vaccines ever developed. However, the host and viral determinants governing YFV-17D attenuation and its potent immunogenicity are still unknown. Here, we analyzed the role of type III interferon (IFN)-mediated signaling, a host immune defense mechanism, in controlling YFV-17D infection and attenuation in different mouse models. We uncovered a critical role of type III IFN-mediated signaling in preserving the integrity of the blood-brain barrier and preventing viral brain invasion. Type III IFN also played a major role in regulating the induction of a potent but balanced immune response that prevented viral evasion of the host immune system. An improved understanding of the complex mechanisms regulating YFV-17D attenuation will provide insights into the key virus-host interactions that regulate host immune responses and infection outcomes as well as open novel avenues for the development of innovative vaccine strategies.

## INTRODUCTION

Arthropod-borne flaviviruses such as dengue virus (DENV), yellow fever virus (YFV), and Zika virus (ZIKV) are a cause of major health concerns worldwide (1–3). With a mortality rate of up to 25% to 50%, YFV infection is one of the most severe flavivirus infections and is responsible for around 200,000 new infections and 30,000 deaths each year (2). The YFV live attenuated strain YFV-17D is one of the safest and most potent vaccines ever developed (4). The vaccination of more than 500 million individuals over the past 80 years has significantly contributed to the prevention of YFV outbreaks (4). However, vaccination coverage has decreased over the last few decades, and recent outbreaks in Africa and South America have highlighted the urgent need for reassessing strategies to control YFV infection worldwide (5).

The intricate interplay between YFV and the human host is poorly understood and limits the design of antiviral therapies. YFV-17D differs from its virulent counterpart, YFV-Asibi, by only 32 amino acids (6). However, despite this close genetic proximity, the differential interactions of YFV and YFV-17D with the human host that determine infection outcome remain unknown. A major limitation in understanding this lies in the restricted human and nonhuman primate tropism of YFV and the lack of proper animal models. Mice are poorly susceptible to YFV infection (7, 8) and hence are not appropriate for fully characterizing the mechanisms that regulate YFV pathogenesis or attenuation.

Interferons (IFNs) are cytokines that play a critical role in the proper induction and maintenance of innate and adaptive immunity (9, 10). There are three known classes of interferons, type I, type II, and type III, which signal through heterodimeric receptors distinct for each IFN type. The type I IFN family, which consists of 17 members, induces signaling through the IFN-α/β receptor (IFN-α/βR). Type I IFN is responsible for the immune response to many viruses, such as lymphocytic choriomeningitis virus (LCMV), Semliki Forest virus, and vesicular stomatitis virus (11), and can inhibit YFV-Asibi and YFV-17D infection in mice (7, 12, 13). The effect of type I IFNs is apparently species specific, as both YFV-17D and virulent YFV can escape the human type I IFN-induced antiviral state (14, 15). Consistently, genetic disruption of type I IFN signaling renders mice highly susceptible to YFV-Asibi infection with a lethal outcome (12). In contrast, YFV-17D extensively replicates and induces an antigen-specific T-cell response in infected IFN-α/βR-deficient mice before ultimately being cleared (16). Consequently, despite the compromised functionality of their innate immune system and an absence of human-specific interactions, mice with impaired type I IFN signaling are regularly used to model YFV pathogenesis and immunogenicity. They represent an interesting complementary tool for evaluating the contribution of host determinants of YFV pathogenesis or YFV-17D attenuation prior to experiments in more relevant models such as macaques or humanized mice.

Type II IFN signaling is mediated through the interaction of IFN-γ—the sole member of this class of IFNs—with the IFN-γ receptor (IFN-γR) (9, 17). IFN-γ is produced by a variety of immune cells, including natural killer (NK) cells, NK T cells, innate lymphoid cells (ILC), and CD4^+^ and CD8^+^ T cells. Type II IFN is critical for combating many bacterial and parasitic and certain viral infections, although type II IFN alone seems to be of limited importance for clearing a majority of viruses (11). Accordingly, mice lacking IFN-γR are not susceptible to virulent YFV infection or to YFV-17D infection (12). However, IFN-γ does play a major role during the late stage of infection when the innate immune system has failed to control the early stages of infection and the adaptive immune response is induced. Indeed, as YFV-17D clearance requires the induction of adaptive immunity in IFN-α/βR^−/−^ mice, infection is lethal in IFN-α/βR^−/−^ IFN-γR^−/−^ mice (12).

Taking the data together, type I IFN-mediated signaling and type II IFN-mediated signaling thus play distinct roles during infection with virulent YFV-Asibi versus live attenuated YFV-17D. Infection with YFV-Asibi, but not with YFV-17D, is lethal when the type I IFN-mediated innate response is impaired. However, during YFV-17D infection, type II IFN signaling is critical for the proper induction of the adaptive immune response and for control of infection if type I IFN responses are depleted or evaded.

Type III IFNs, discovered in 2003 (18), consist of a group of four cytokines (IFN-λ1, IFN-λ2, IFN-λ3, and IFN-λ4) which signal through the IFN-λ receptor (IFN-λR), comprised of the IFN-λR1 and the IL-10R2 chains. While the type I and type III IFN signaling pathways share some features (19), there is accumulating evidence for the existence of a broader spectrum of IFN-λ-mediated immune functions than initially thought (20, 21). In contrast to IFN-α/βR, which is broadly expressed among all nucleated cells, IFN-λR is preferentially expressed on epithelial surfaces (19, 20). Thus, type III IFN-mediated signaling conceivably plays a unique role in protecting epithelial barriers by the activity of a more localized and balanced immune response. IFN-λ has also been shown to display important and specific roles in the modulation of adaptive immune responses during cancer, viral infection, and autoimmune diseases (20).

In the context of flavivirus infection, IFN-λR^−/−^ mice are notably more permissive to infection with West Nile virus (WNV), a neurotropic flavivirus. IFN-λ appeared to be critical for tightening the blood-brain barrier (BBB) and restricting viral neuroinvasion (22). However, the contribution of type III IFN signaling during YFV infection has not yet been characterized.

In this study, we aimed to address the role of type III IFN in controlling YFV-17D attenuation. Fully immunocompetent (wild-type [WT]) mice were largely resistant to YFV-17D infection, and depletion of type III IFN signaling did not lead to any significant pathogenesis phenotype. Indeed, IFN-λR-deficient mice (IFN-λR^−/−^) rapidly cleared viral infection in a manner similar to that seen with WT mice. IFN-α/βR^−/−^ mice exhibited prolonged viremia following YFV-17D infection and induction of adaptive immune responses but did not succumb to infection. In contrast, YFV-17D infection was lethal in IFN-α/βR λR doubly deficient mice (IFN-α/βR^−/−^ IFN-λR^−/−^), highlighting a critical role for type III IFN in controlling YFV-17D infection in the context of extensive viral replication. α/βR^−/−^ λR^−/−^ mice displayed impaired T-cell activation, excessive proinflammatory secretion as well as increased BBB permeability. Taking the data together, type III IFN is likely a key component that preserves viral attenuation and maintains a host environment conducive to the induction of potent adaptive immunity.

## RESULTS

### **IFN-α/βR**^−/−^ **λR**^−/−^ **mice are hypersusceptible to YFV-17D infection.**

**FIG 1:**
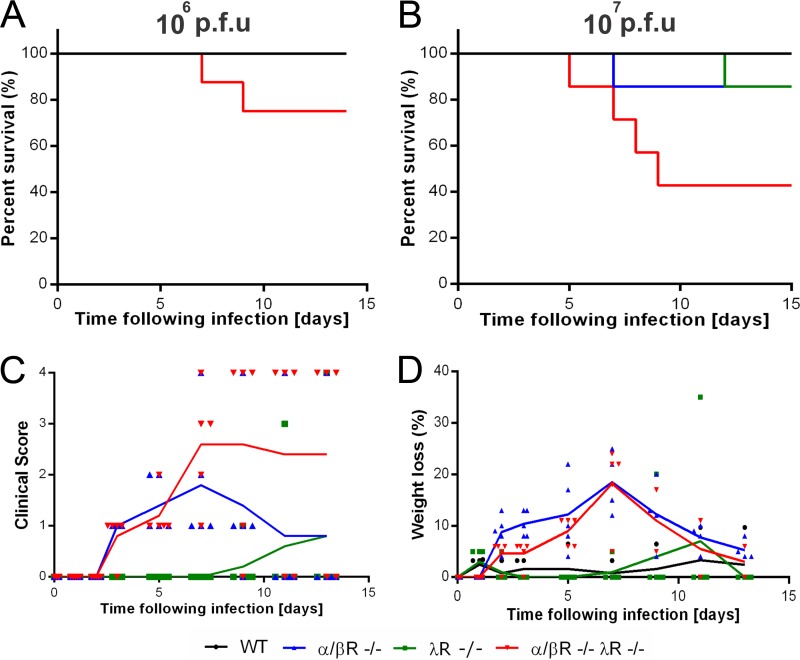
α/βR^−/−^ λR^−/−^ mice are hypersusceptible to YFV-17D infection. (A and B) Survival of WT (black), λR^−/−^ (green), αβR^−/−^ (blue), and αβR^−/−^ λR^−/−^ (red) mice following YFV-17D infection with 10^6^ PFU (A) or 10^7^ PFU (B) (*n* = 5 to 7 per group). (C and D) Clinical score (C) and weight loss (D) of WT (black), λR^−/−^ (green), αβR^−/−^ (blue), and αβR^−/−^ λR^−/−^ (red) mice following YFV-17D infection with 10^7^ PFU. The solid line represents the mean for each time point and group of animals (*n* = 5).

We infected WT, αβR^−/−^, λR^−/−^, and αβR^−/−^ λR^−/−^ mice with YFV-17D (10^6^ PFU) and monitored the survival rates of the mice over a 15-day course of infection. αβR^−/−^ λR^−/−^ mice exhibited increased susceptibility to viral infection (30% mortality rate) in comparison to all the other mouse strains tested (Fig. 1A). Escalating the dose of YFV-17D (10^7^ PFU) increased the mortality rate of αβR^−/−^ λR^−/−^ mice (survival rate at 9 days postinfection, 40%), but more than 90% of the αβR^−/−^ and λR^−/−^ survived the viral infection (Fig. 1B). Infected with 10^7^ PFU YFV-17D, both αβR^−/−^ and αβR^−/−^ λR^−/−^ mice displayed clinical manifestations of disease, as well as significant weight loss and temperature drop, between days 5 and 10 postinfection, although the WT mice and λR^−/−^ mice did not exhibit any clinical signs of disease (Fig. 1C and D; see also Fig. S1 in the supplemental material). αβR^−/−^ mice progressed to clinically apparent disease that reached a peak around day 7 postinfection but recovered over time. αβR^−/−^ λR^−/−^ mice exhibited similar initial signs of morbidity, but the symptoms worsened and the animals became moribund or died (Fig. 1C). Hence, although αβR^−/−^ mice and αβR^−/−^ λR^−/−^ mice seemed to be similarly susceptible to YFV-17D at early time points postinfection, most of the αβR^−/−^ λR^−/−^ mice, in contrast to the αβR^−/−^ mice, were not able to recover from infection. Taken together, our data suggest that IFN-λ likely plays a role in preventing the worsening of YFV-17D-induced disease at later stages of infection.

10.1128/mBio.00819-17.1 FIG S1  Body temperature variations during YFV-17D infection. Download FIG S1, DOCX file, 0.1 MB. Copyright © 2017 Douam et al. 2017 Douam et al. This content is distributed under the terms of the Creative Commons Attribution 4.0 International license.

### Type III IFN-mediated signaling prevents spread of YFV-17D to the central nervous system (CNS).

**FIG 2:**
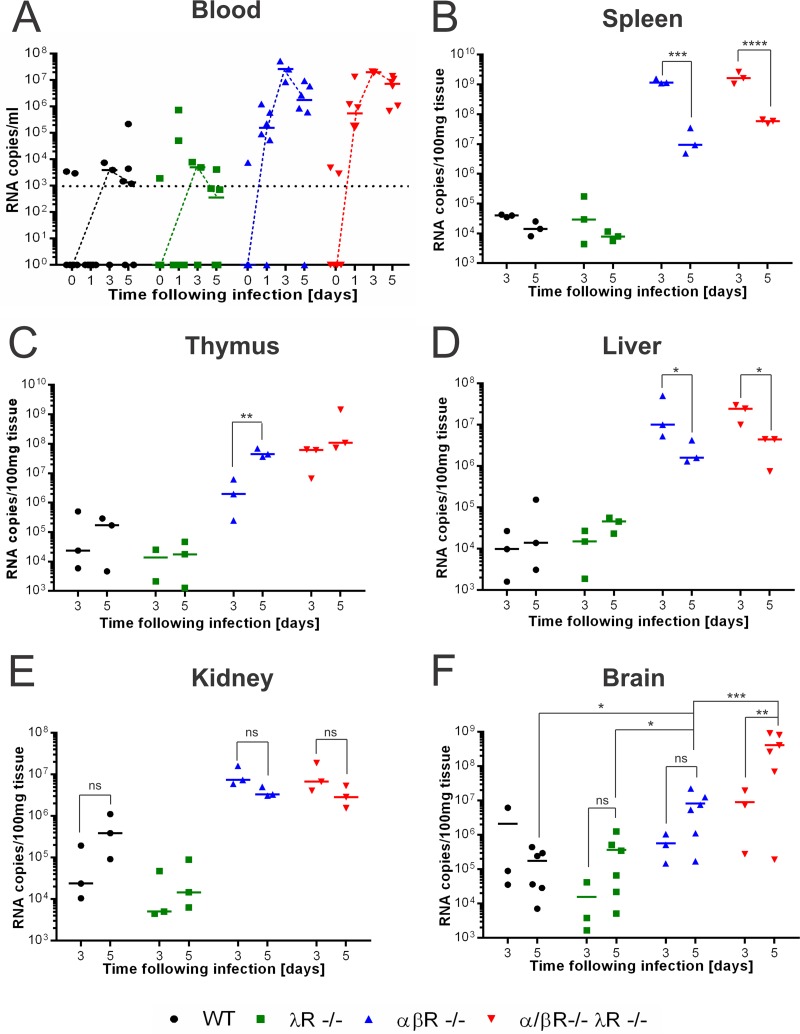
Severe neuroinvasion in αβR^−/−^ λR^−/−^ mice. (A) Serum viremia of WT (black), λR^−/−^ (green), αβR^−/−^ (blue), and αβR^−/−^ λR^−/−^ (red) mice prior to YFV-17D infection and at days 1, 3, and 5 postinfection (10^7^ PFU). Positive-strand RNA copies per milliliter were quantified by RT-qPCR. Nonhorizontal dotted lines link the medians for the mouse models. The limit of detection (horizontal dotted line) is shown (*n* = 3 to 6 per group). (B to F) Viral loads within spleen (B), thymus (C), liver (D), kidney (E), and brain (F) tissue of WT (black), λR^−/−^ (green), αβR^−/−^ (blue), and αβR^−/−^ λR^−/−^ (red) mice at days 3 and 5 following YFV-17D infection (10^7^ PFU). Positive-strand RNA copies were quantified by RT-qPCR and normalized to 100 mg of tissue. Medians (dotted lines) are shown. The limit of detection is represented by the lowest *y* axis value (10^3^ RNA copies/100 mg tissue) (*n* = 3 to 6 per group). *, *P* < 0.05; **, *P* < 0.01; ***, *P* < 0.001; ****, *P* < 0.0001; ns, nonsignificant.

Next, we aimed to identify the virologic event(s) specific to αβR^−/−^ λR^−/−^ mice and not to αβR^−/−^ or αβR^−/−^ mice. We quantified viral RNA in the serum of WT, αβR^−/−^, λR^−/−^, and αβR^−/−^ λR^−/−^ mice at days 0, 1, 3, and 5 postinfection. As expected, the WT and λR^−/−^ mice exhibited low levels of viremia throughout this period, likely resulting in complete viral clearance (Fig. 2A). In contrast, αβR^−/−^ and αβR^−/−^ λR^−/−^ mice were highly viremic during the first 5 days postinfection (Fig. 2A), demonstrating the critical role of type I IFN in controlling early viral replication and consistent with previous findings (7, 12, 13). IFN-λ did not appear to have any impact on peripheral viral replication even when type I IFN signaling was abrogated. Consequently, we quantified the YFV-17D viral load in more localized tissue compartments of these mouse strains.

Evidence of viral replication was found in all tested tissues (spleen, thymus, liver, kidney, and brain) across all cohorts (Fig. 2B to F). While viral RNA levels decreased to below the limit of detection around day 5 postinfection in the sera of WT and λR^−/−^ mice, YFV-17D persisted longer (up to 5 days) in other tissues, as previously reported in YFV-17D-infected WT mice (23). This longer persistence likely preceded viral clearance, as these mice survived up to 15 days postinfection and no significant increase in viral RNA was observed in the tested tissues between days 3 and 5 postinfection.

In the spleen and thymus (Fig. 2B and C), two lymphoid tissues, viral replication was similarly enhanced in the αβR^−/−^ and αβR^−/−^ λR^−/−^ mice in comparison to the WT and λR^−/−^ mice. This trend was also observed in the kidney and liver (Fig. 2D and E), two visceral tissues where YFV can replicate. The viral load remained stable or decreased in the spleen, liver, and kidney of αβR^−/−^ and αβR^−/−^ λR^−/−^ mice between days 3 and 5 postinfection, suggesting that the immune response might control viral replication in these tissues. Rare cases of neurologic adverse effects have been observed in YFV-17D-infected rhesus macaques, human vaccinees (2, 24–26), and αβR^−/−^ mice (7). We found that the viral load in the brains of αβR^−/−^ mice and αβR^−/−^ λR^−/−^ mice was more elevated than in those of WT and λR^−/−^ mice at day 5 postinfection (Fig. 2F). However, the viral load in the brains of αβR^−/−^ λR^−/−^ mice was significantly higher than in those of αβR^−/−^ mice at day 5 postinfection, suggesting a more prominent viral neuroinvasion of the brains of αβR^−/−^ λR^−/−^ mice. An increase in viral load was observed between days 3 and 5 only in αβR^−/−^ λR^−/−^ mice, suggesting active viral replication in the brain of this mouse strain but not in the other three groups of mice (Fig. 2F). No differences in viral load were detected between the WT and λR^−/−^ mice at day 3 or day 5 postinfection. Taken together, our results suggest that type III IFN signaling is a critical regulator that can prevent lethal viral neuroinvasion during extensive YFV-17D infection.

### **αβR**^−/−^ **λR**^−/−^ **mice exhibit more-prominent brain histopathological manifestations.**

**FIG 3:**
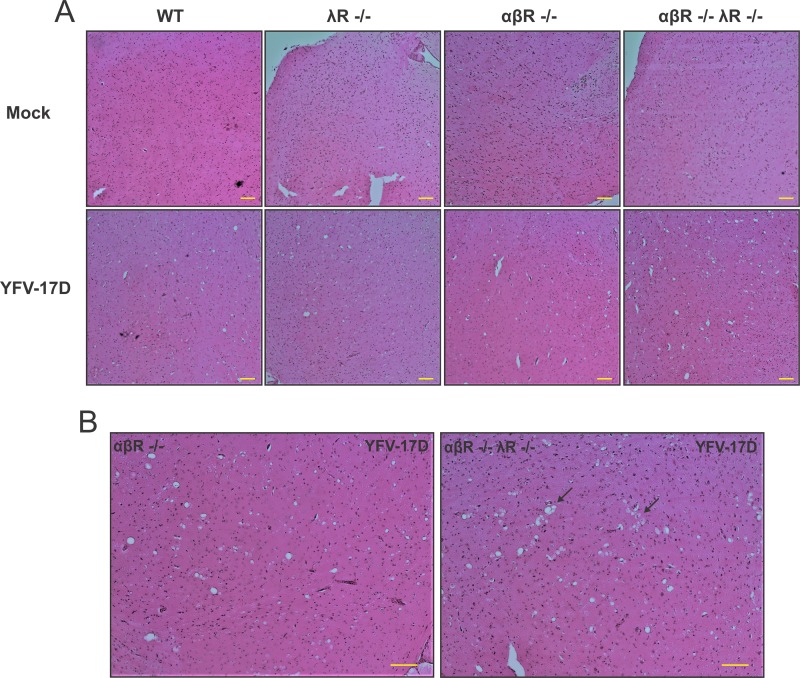
Evidence for virally induced brain tissue damage in αβR^−/−^ λR^−/−^ mice. (A) Hematoxylin and eosin (H&E) staining of mouse brain cortex (magnification, ×20) tissue sections from noninfected (Mock) and infected WT, λR^−/−^, αβR^−/−^, and αβR^−/−^ λR^−/−^ mice at day 5 following YFV-17D infection (10^7^ PFU). Pictures of brain cortex from infected αβR^−/−^ and αβR^−/−^ λR^−/−^ mice displaying representative spongiosis are shown enlarged in panel B. For each experimental condition (noninfected or infected), six tissue sections from three biological replicates (three animals) were examined. Histopathological manifestations observed in infected animal tissues were absent from all examined noninfected animals; results are representative of three biological replicates for a given type of tissue. Arrows indicate representative areas with severe spongiosis and multivacuolar structures. Scale bars (100 µm) are indicated for each picture.

We then aimed to identify whether extensive YFV-17D replication in the brain of αβR^−/−^ λR^−/−^ mice induced significant histopathological manifestations that could be related to the increased mortality of these mice. YFV-17D neurovirulence in rhesus macaques has been characterized by the presence of perivascular cuffs and infiltrating mononuclear inflammatory cells in the brain cortex, leading to the destruction of neuronal cells and neuronophagia (24). At day 5 postinfection, all infected brains, regardless of the mouse model, exhibited signs of neuropil vacuolization (or spongiosis) in the brain cortex consistent with all mouse models exhibiting brain viremia (Fig. 3A). This histopathological manifestation has been previously reported during WNV brain invasion (27, 28) and other neurovirulent flavivirus infections (29). However, spongiosis appeared to be more prominent in αβR^−/−^ λR^−/−^ mice (Fig. 3), hence suggesting more significant damage of the cortex in these mice.

In contrast, despite significant viral replication, we did not observe major histopathological changes in the liver of infected αβR^−/−^ and αβR^−/−^ λR^−/−^ mice at day 5 postinfection. No significant acute/chronic inflammations, hepatocellular changes, fibrosis, or necrosis was observed in these two mouse models compared to noninfected mice or infected WT or λR^−/−^ mice (data not shown). This adds to the evidence that viral replication in the liver is not directly involved in the pathogenesis observed in αβR^−/−^ λR^−/−^ mice, as suggested by similar levels of viral replication in αβR^−/−^ mice.

### **Increased permeability of the blood-brain barrier in IFN-α/βR**^−/−^ **λR**^−/−^ **mice during YFV-17D infection.**

IFN-αβ and IFN-λ have previously been reported to regulate the permeability of the BBB during WNV infection (22, 30). Thus, we hypothesized that YFV-17D neuroinvasion might also be aided by changes in the permeability of the BBB of αβR^−/−^ λR^−/−^ mice during infection. To directly test this hypothesis, we infected WT, αβR^−/−^, λR^−/−^, and αβR^−/−^ λR^−/−^ mice with YFV-17D (10^7^ PFU) and injected them intravenously with Evans blue dye just prior to sacrifice and tissue collection. We then measured the dye absorbance into the brain as well as into additional tissue controls to quantify potential increases in BBB permeability.

**FIG 4:**
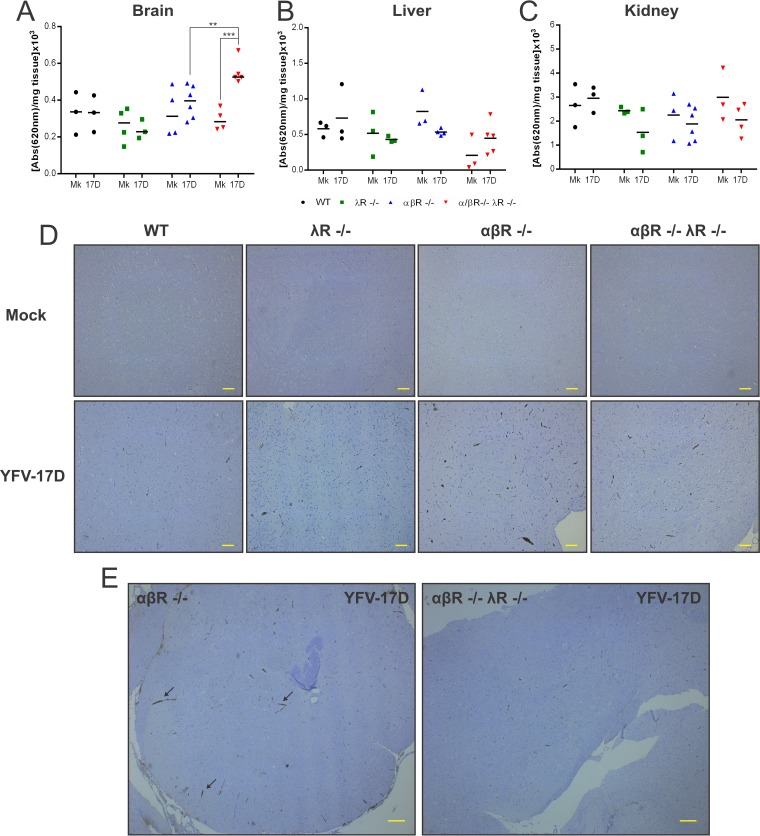
Increased permeability of the blood-brain barrier in αβR^−/−^ λR^−/−^ mice upon YFV-17D infection. Evans blue dye absorbance (620 nm) of indicated tissues (A, brain; B, liver; C, kidney) isolated from noninfected (mock; Mk) or infected (YFV-17D) WT (black), λR^−/−^ (green), αβR^−/−^ (blue), and αβR^−/−^ λR^−/−^ (red) mice at day 5 following YFV-17D infection (10^7^ PFU). The values representing the absorbance were normalized to tissue mass. Evans blue was administered by intravenous injection 30 min prior to sacrifice (*n* = 3 to 6 per group). **, *P* < 0.01; ***, *P* < 0.001. (D) CD3 staining of mouse brain cortex tissue sections from noninfected (Mk) and infected WT, λR^−/−^, αβR^−/−^, and αβR^−/−^ λR^−/−^ mice at day 5 following YFV-17D infection (10^7^ PFU) (magnification, ×20; scale bar, 100 µm). (E) Representative pictures of brain cortex from infected αβR^−/−^ and αβR^−/−^ λR^−/−^ mice are displayed with a ×4 magnification (scale bar = 200 µm). For each experimental condition (noninfected or infected), six tissue sections from three biological replicates (three animals) were examined. Histopathological manifestations observed in infected animal tissues were absent from all examined noninfected animals; results are representative of three biological replicates. Arrows indicate representative areas with severe lymphocyte infiltration.

While brains from WT, λR^−/−^, and αβR^−/−^ mice did not become more permeable to the Evans blue dye following infection, there was a statistically significant increase in the BBB permeability of αβR^−/−^ λR^−/−^ mice (Fig. 4A). As controls, the permeability of the liver and kidney remained unchanged upon infection (Fig. 4B and C). Taken together, our results suggest that type III IFN signaling is an important regulator in preserving BBB integrity during extensive YFV-17D infection.

Increased BBB permeability is commonly associated with enhanced lymphocyte infiltration in the central nervous system of WT mice during WNV infection (31). Histological analysis of brain tissue sections from noninfected and infected animals revealed that all mouse strains displayed an increase in CD3^+^ T-cell levels in the brain cortex following YFV-17D infection (Fig. 4D). However, T-cell infiltration following YFV-17D infection was more extensive in αβR^−/−^ mice than in WT mice, λR^−/−^ mice, and αβR^−/−^ λR^−/−^ mice (Fig. 4D and E). αβR^−/−^ λR^−/−^ mice seemed to have a level of T-cell infiltration similar to that seen with the WT and λR^−/−^ mice despite extensive viral neuroinvasion and enhanced BBB permeability (Fig. 4D). These results suggest that the T-cell-mediated adaptive immune response might be hampered in αβR^−/−^ λR^−/−^ mice, hence enhancing viral immune evasion and neuroinvasion.

### Type III IFN signaling impacts immune subset frequencies during YFV-17D infection.

**FIG 5:**
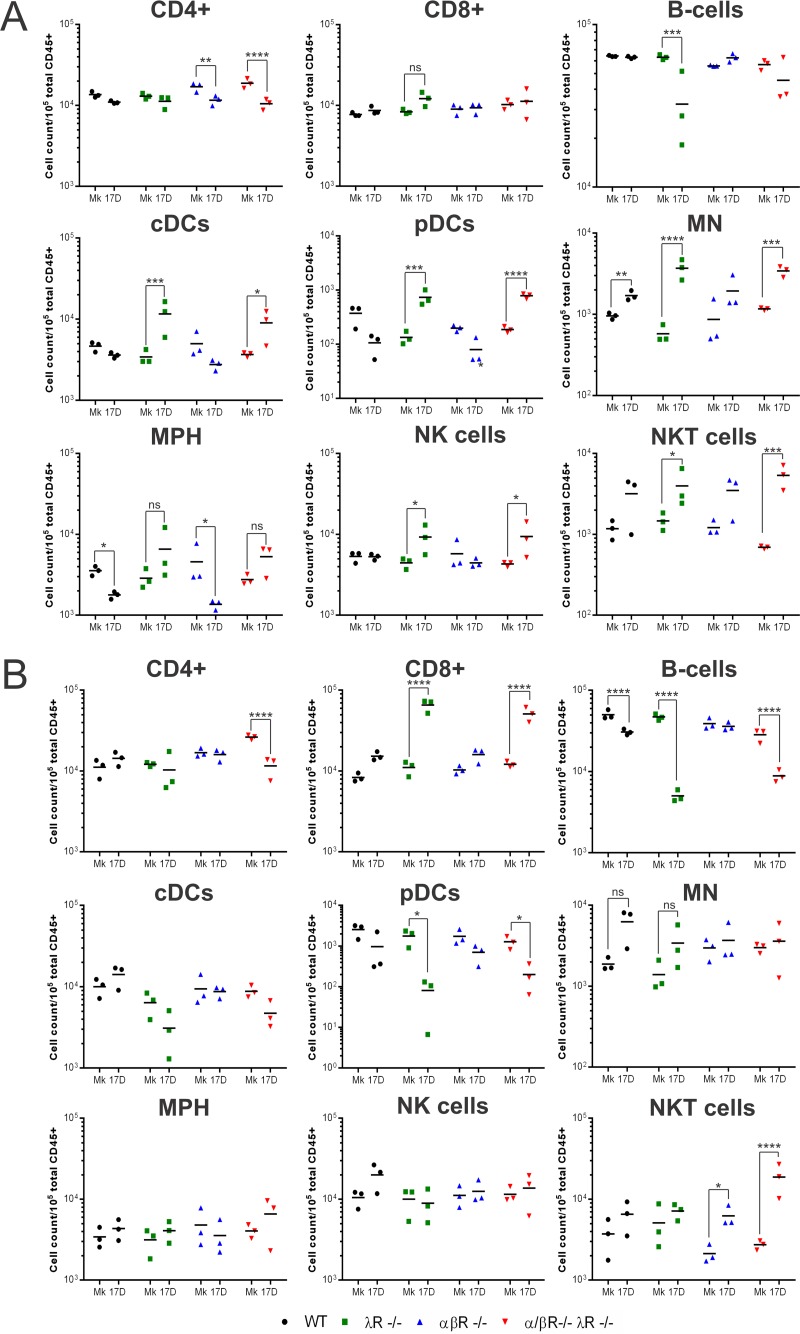
Disruption of type III IFN-mediated signaling impacts immune cell lineage proliferation. Total numbers of the indicated immune cell subsets (total cell count per 10^5^ total murine CD45^+^ cells) in the spleen (A) and liver (B) of noninfected (Mk, mock) or infected (YFV-17D) WT (black), λR^−/−^ (green), αβR^−/−^ (blue), and αβR^−/−^ λR^−/−^ (red) mice at day 5 following YFV-17D infection (10^7^ PFU) are shown. Statistical analysis shows significant differences between the results determined under noninfected (Mk, mock) and infected (YFV-17D) conditions for each mouse strain and cell lineage (*n* = 3 per group). *, *P* < 0.05; **, *P* < 0.01; ***, *P* < 0.001; ****, *P* < 0.0001; ns, nonsignificant. CD4^+^, CD4^+^ T cells; CD8^+^, CD8^+^ T cells; cDCs, conventional dendritic cells; pDCs, plasmacytoid dendritic cells; MN, monocytes; MPH, macrophages; NK cells, natural killer cells; NKT cells, natural killer T cells.

We then evaluated whether a lack of type III IFN signaling could impact the dynamics of immune subsets in response to YFV-17D infection, hence favoring virus immune evasion and subsequent neuroinvasion. In the spleen, depletion of type III IFN signaling alone resulted in significantly higher numbers of conventional dendritic cells (cDCs), plasmacytoid dendritic cells (pDCs), and monocytes (MN) upon YFV-17D infection, as well as a decrease in the levels of B cells (Fig. 5A). Moreover, the number of spleen-resident macrophages decreased upon infection in WT and αβR^−/−^ mice but not in λR^−/−^ and αβR^−/−^ λR^−/−^ mice. Depletion of type I IFN alone appeared to impact the numbers of CD4^+^ T cells, which were reduced in the spleen upon infection. Consistently, similar changes in CD4^+^ T-cell numbers were also found in αβR^−/−^ λR^−/−^ mice. Many of the cell lineage phenotypes observed in λR^−/−^ mice were observed in αβR^−/−^ λR^−/−^ mice but not in αβR^−/−^ mice (Fig. 5A), highlighting the critical role of type III IFN in regulating the numbers of immune cell subsets during YFV-17D infection. Additionally, we observed significant (up to 8-fold) increases in the numbers of spleen-resident NK and NKT cells (Fig. 5A) in αβR^−/−^ λR^−/−^ mice.

CD8^+^ T cells were significantly increased in number and B cells and pDCs were decreased in number in the livers of λR^−/−^ mice (Fig. 5B). Similar fluctuations in these cellular subsets were observed in the livers of αβR^−/−^ λR^−/−^ mice but not in those of αβR^−/−^ mice, strengthening the idea of a critical role of type III IFN in regulating the expansion and/or migration of these subsets. αβR^−/−^ λR^−/−^ mice also specifically exhibited a significant decrease in levels of liver CD4^+^ T cells as well as an important increase in levels of liver NKT cells greater than that shown by the liver NKT cells observed in αβR^−/−^ mice (Fig. 5B).

In the blood, two major phenotypes specifically induced by the depletion of type III IFN in WT and αβR^−/−^ mice were observed. First, the number of peripheral B cells decreased upon infection in λR^−/−^ mice and αβR^−/−^ λR^−/−^ mice but not in WT and αβR^−/−^ mice (Fig. S2A). Second, genetically abrogating type III IFN signaling appeared to affect the number of peripheral pDCs upon infection, as the number increased only in the WT and αβR^−/−^ mice (Fig. S2A).

10.1128/mBio.00819-17.2 FIG S2  Type III IFN-mediated signaling impacts peripheral cell lineage frequencies and liver T-cell activation. Download FIG S2, DOCX file, 0.4 MB. Copyright © 2017 Douam et al. 2017 Douam et al. This content is distributed under the terms of the Creative Commons Attribution 4.0 International license.

Taken together, our data provide evidence that blunting type III IFN signaling distorts the number of immune cell subsets during YFV-17D infection. The absence of type III IFN signaling in αβR^−/−^ λR^−/−^ mice could thus significantly perturb the correct immune balance and coordination, consequently stimulating virus evasion of the host immune response.

### Depletion of type III IFN signaling impairs T-cell activation during YFV-17D infection.

Depletion of type I IFN allows YFV-17D to replicate extensively in spite of innate immune defense barriers, hence favoring the induction of adaptive immunity. YFV-17D infection was previously shown to be cleared from αβR^−/−^ mice by a T-cell-specific response (16). As IFN-λ appears to affect cell lineage proliferation upon YFV-17D infection and has previously been shown to regulate adaptive immune responses (20, 32, 33), we sought to identify whether impaired T-cell activation could contribute to immune evasion and increased pathogenesis in αβR^−/−^ λR^−/−^ mice.

For this purpose, we analyzed the changes in expression of multiple activation/exhaustion markers on the surface of CD4^+^ and CD8^+^ T cells in our different cohorts during infection (day 5 postinfection). We examined the following: CD62L and CCR7, two receptors highly expressed on naive and memory T cells that mediate the preferential homing of T cells into lymphoid organs (34, 35); CD45RA, a T-cell receptor signaling molecule highly expressed in naive T cells but absent in central and effector memory T cells following antigen experience (35, 36); CD127, a part of the IL-7 receptor critical for T-cell homeostasis and survival that is downregulated upon expansion of effector T cells (37); CD27 and CD28, two costimulatory receptors critical for T-cell activation that increase in expression upon activation and are then downregulated in effector and effector memory T cells (38); PD1, an inhibitory costimulator receptor that is upregulated upon T-cell activation and highly expressed on exhausted T cells (38); and CD44, a cell adhesion molecule that stimulates T-cell activation and proliferation and is highly expressed on effector and memory T cells (39).

**FIG 6:**
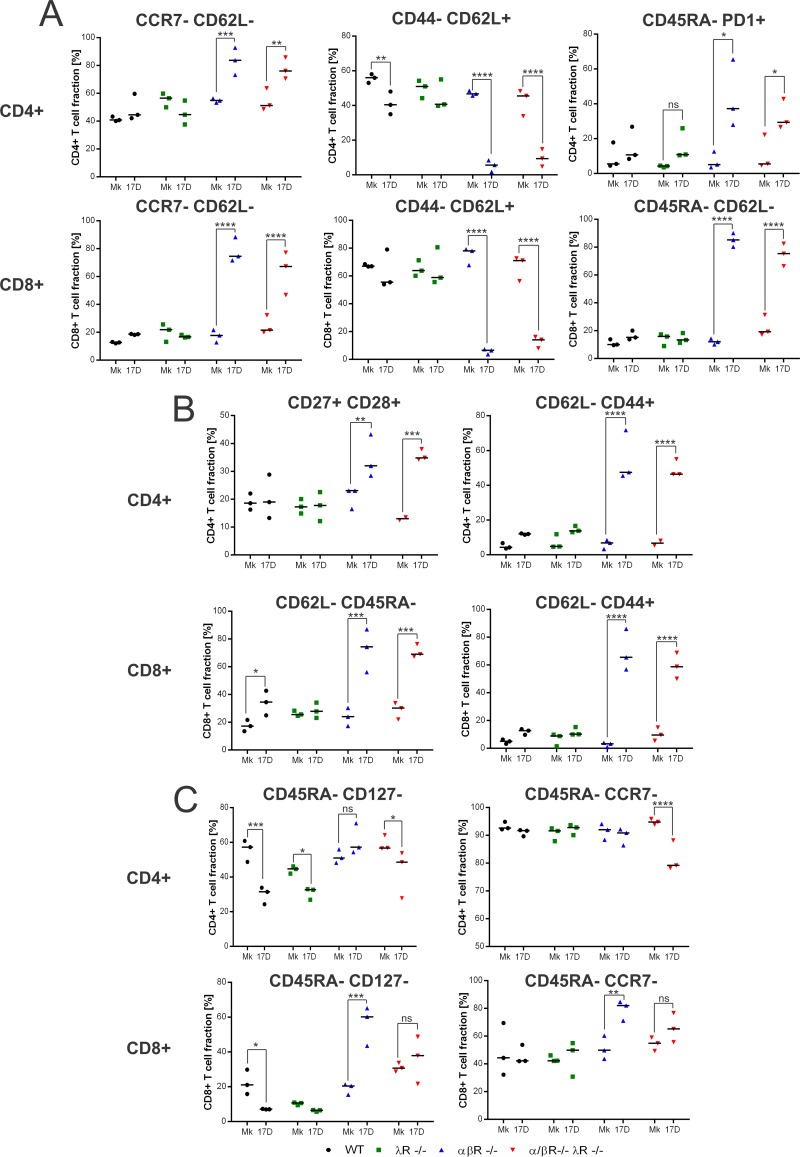
Abrogation of type III IFN-mediated signaling impairs T-cell activation. Characterization of the activation phenotype of CD3^+^ CD4^+^ and CD3^+^ CD8^+^ T-cell populations in the spleen (A and C) and blood (B) of noninfected (Mk, mock) or infected (YFV-17D) WT (black), λR^−/−^ (green), αβR^−/−^ (blue), and αβR^−/−^ λR^−/−^ (red) mice at day 5 following YFV-17D infection (10^7^ PFU) was performed. Immune cell activation was characterized by quantifying the expression of activation or exhaustion markers in CD4^+^ or CD8^+^ T-cell populations using a set of eight markers (CD45RA, CD127, CCR7, CD44, CD62L, PD1, CD27, and CD28). Panel A and C delineate spleen-resident T-cell activation profiles that are found to be similar or different respectively between αβR^−/−^ and αβR^−/−^ λR^−/−^ mice. Statistical analysis showed significant differences between the results determined under noninfected (Mk, mock) and infected (YFV-17D) conditions for each mouse model and cell lineage (*n* = 3 per group). *, *P* < 0.05; **, *P* < 0.01; ***, *P* < 0.001; ****, *P* < 0.0001; ns, nonsignificant.

The spleens of αβR^−/−^ and αβR^−/−^ λR^−/−^ mice exhibited an increase in the levels of CCR7^−^ CD62L^−^ CD4^+^ and CD8^+^ T cells upon infection (Fig. 6A). Moreover, a decrease in the levels of CD44^−^ CD62L^+^ CD4^+^ and CD8^+^ T cells and an increase in the levels of CD45RA^−^ CD62L^−^ CD8^+^ T cells were observed in αβR^−/−^ and αβR^−/−^ λR^−/−^ mice (Fig. 6A). Taken together, these data suggest that YFV-17D infection in αβR^−/−^ and αβR^−/−^ λR^−/−^ mice primes effector CD4^+^ and CD8^+^ T cells, which can then migrate to peripheral tissues. The observed increase in the levels of CD45RA^−^ PD1^+^ CD4^+^ T cells and CD45RA^−^ CD62L^−^ CD8^+^ T cells in αβR^−/−^ and αβR^−/−^ λR^−/−^ mice supports this conclusion (Fig. 6A). In the liver, increases in the number of effector CD62L^−^ CD44^+^ CD4^+^ T cells and CD8^+^ T cells were also observed (Fig. S2B). In the blood, more-pronounced CD27 and CD28 expression among CD4^+^ T cells in αβR^−/−^ and αβR^−/−^ λR^−/−^ mice underscored a potential CD4^+^ T-cell activation, and an increase in the numbers of CD62L^−^ CD44^+^ CD4^+^ T cells suggested the development of peripheral effector CD4^+^ T-cell populations (Fig. 6B). Similarly, peripheral CD62L^−^ CD45RA^−^ and CD62L^−^ CD44^+^ CD8^+^ T cells were more prominent in αβR^−/−^ and αβR^−/−^ λR^−/−^ mice, highlighting an increase in the effector CD8^+^ T-cell population in the periphery (Fig. 6B). However, only partial T-cell activation was found in αβR^−/−^ λR^−/−^ mice. Although the CD45RA^−^ CD127^−^ and CD45RA^−^ CCR7^−^ CD4^+^ T-cell populations were stable upon YFV-17D infection in the spleen of αβR^−/−^ mice, these populations experienced a significant decrease in the spleen of αβR^−/−^ λR^−/−^ mice (Fig. 6C). In parallel, the levels of spleen-resident CD45RA^−^ CD127^−^ and CD45RA^−^ CCR7^−^ CD8^+^ T-cell populations increased in αβR^−/−^ mice but remained stable in αβR^−/−^ λR^−/−^ mice (Fig. 6C).

We observed a decrease in CD45RA^−^ CD127^−^ CD4^+^ T cells in the liver of αβR^−/−^ λR^−/−^ mice but not in the liver of αβR^−/−^ mice (Fig. S2C). Such a decrease was also observed in WT and λR^−/−^ mice, although these mice did not experience extensive viral replication like that seen with αβR^−/−^ λR^−/−^ mice, suggesting a nonappropriate T-cell phenotype in the liver of αβR^−/−^ λR^−/−^ mice. Finally, fewer CD8^+^ T cells displayed an activated (CD45RA^−^ CD127) phenotype in αβR^−/−^ λR^−/−^ mice than in αβR^−/−^ mice (Fig. S2C).

Taken together, our results highlight that depletion of type III IFN signaling negatively impacts T-cell activation in the context of extensive viral replication.

### **Elevated concentration of IFN-γ in IFN-α/βR**^−/−^ **λR**^−/−^ **mice upon YFV-17D infection.**

**FIG 7:**
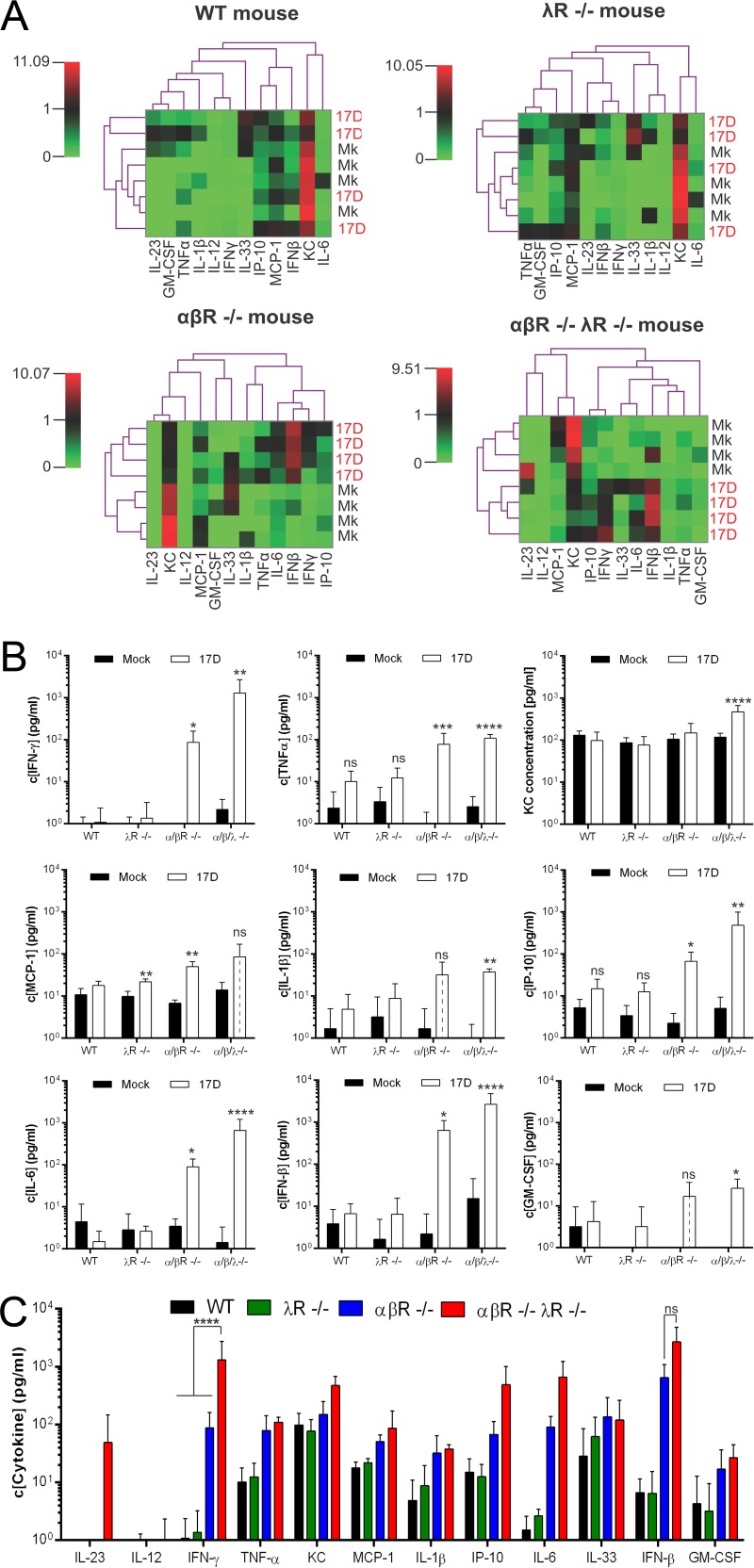
Cytokine profiles induced by YFV-17D infection in type I and type III IFN-deficient mice. (A) Heat maps modelling the concentration changes of 12 cytokines in the blood of noninfected (Mk, mock) and infected (YFV-17D) wild-type (WT), λR^−/−^, αβR^−/−^, and αβR^−/−^ λR^−/−^ mice at day 5 postinfection (10^7^ PFU). (B) Concentrations of nine cytokines in the blood of noninfected (Mock) or infected (YFV-17D) WT, λR^−/−^, αβR^−/−^, and αβR^−/−^ λR^−/−^ mice at day 5 postinfection (10^7^ PFU). Statistical analysis showed significant differences between the results determined under noninfected (Mk, mock) and infected (YFV-17D) conditions for each mouse strain (means ± standard deviations [SD]; *n* = 3 per group). *, *P* < 0.05; **, *P* < 0.01; ***, *P* < 0.001; ****, *P* < 0.0001; ns, nonsignificant. (C) Differences in blood cytokine concentrations between infected WT (black), λR^−/−^ (green), αβR^−/−^ (blue), and αβR^−/−^ λR^−/−^ (red) mice at day 5 post-YFV-17D infection (10^7^ PFU) (means ± SD; *n* = 3 per group). ****, *P* < 0.0001; ns, nonsignificant.

Increased BBB permeability was not observed in noninfected αβR^−/−^ λR^−/−^ mice, suggesting that additional host or viral factors might contribute to this phenotype upon infection. Th1 proinflammatory cytokines such as IFN-γ have previously been reported to increase BBB permeability during virus infection (30, 40). Thus, we sought to determine the levels of proinflammatory cytokines in our different mouse cohorts upon YFV-17D infection. We analyzed the serum concentration of 12 cytokines (interleukin-23 [IL-23], IL-12, IFN-γ, tumor necrosis factor alpha [TNF-α], keratinocyte chemoattractant [KC], monocyte chemoattractant protein-1 [MCP-1], IL-1β, interferon gamma-induced protein 10 [IP-10], IL-6, IL-33, IFN-β, and granulocyte-macrophage colony-stimulating factor [GM-CSF]) in WT, λR^−/−^, αβR^−/−^, and αβR^−/−^ λR^−/−^ mice before and at day 5 postinfection. In contrast to WT and λR^−/−^ mice, infection of αβR^−/−^ and αβR^−/−^ λR^−/−^ mice induced significant cytokine concentration changes in the blood. Indeed, the cytokine profiles of all infected αβR^−/−^ and αβR^−/−^ λR^−/−^ mice distinctly clustered separately from the profiles of noninfected animals (Fig. 7A). Of the 12 cytokines that we quantified, 9 (Fig. 7B) exhibited a significant increase upon infection in αβR^−/−^ and/or αβR^−/−^ λR^−/−^ mice whereas only 1 (MCP-1) exhibited a slight increase upon infection of λR^−/−^ mice. No increases were detected in infected WT mice.

The significant elevation in the concentration of multiple cytokines in αβR^−/−^ and/or αβR^−/−^ λR^−/−^ mice was associated with T-cell activation and the presence of persistent viral replication. An increase in serum IP-10 levels has been associated with YFV-17D replication in YFV-17D vaccinees and in other αβR^−/−^ mouse models (7, 41). Increases in GM-CSF, IL-1β, and KC levels were observed only in αβR^−/−^ λR^−/−^ mice upon infection (Fig. 7B), but the concentrations were not significantly different from those observed in αβR^−/−^ mice following infection.

Among all the cytokines tested, proinflammatory cytokine IFN-γ was the only one that displayed a significantly higher concentration in the serum of αβR^−/−^ λR^−/−^ mice than in the serum of αβR^−/−^ mice and the two other mouse models (Fig. 7C). This result suggests that high serum levels of IFN-γ could potentially contribute to the pathogenesis phenotype observed in αβR^−/−^ λR^−/−^ mice via perturbation of host immune responses and BBB integrity. Taken together, our results suggest that type III IFN might play an important role in regulating the proinflammatory/anti-inflammatory cytokine balance during YFV-17D infection.

## DISCUSSION

**FIG 8:**
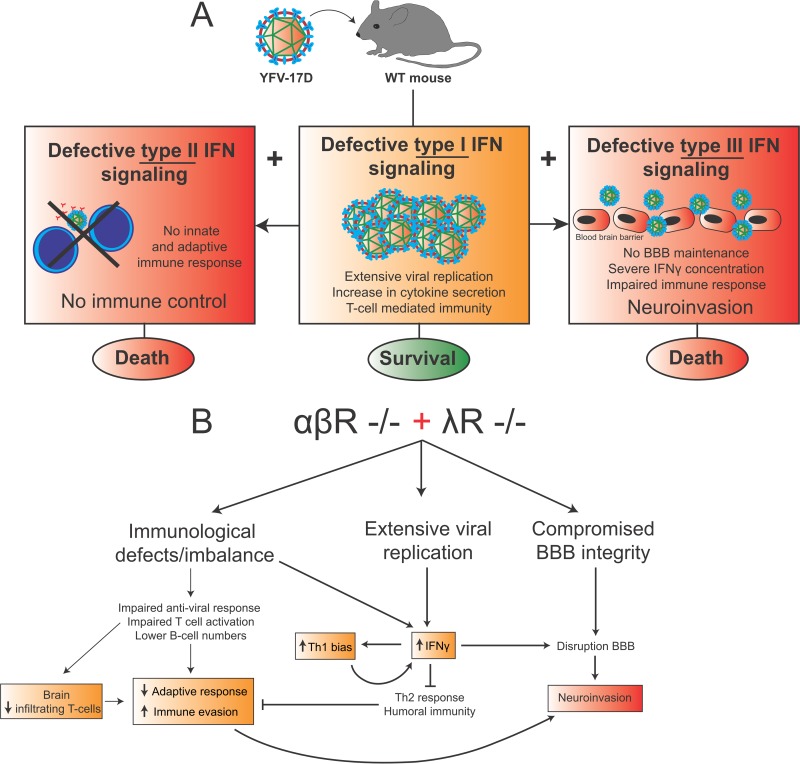
Impact of type III IFN signaling on YFV-17D infection. (A) Schematic representation of the impact of the depletion of type I (middle), type I and type II (left), or type I and type III (right) IFN-mediated signaling in mouse during YFV-17D infection. Although type I IFN-mediated signaling depletion led to the induction of an adaptive immune response and survival, the dual depletion of both type I and type II or both type I and type III IFN-mediated signaling led to severe pathogenesis and death through distinct mechanisms. (B) Proposed pathogenesis model in αβR^−/−^ λR^−/−^ mice infected by YFV-17D. We suggest that αβR^−/−^ λR^−/−^ mice display three distinct pathogenesis features: immunological defects, extensive viral replication, and compromised BBB integrity. We propose a model in which these features are connected and contribute as a whole to viral neuroinvasion.

The mechanisms governing the strong immunogenicity of YFV-17D remain incompletely understood. Induction of a protective immune response to YFV-17D is likely dependent on the potent ability of YFV-17D to replicate in human cells (23). During the induction of protective immunity in response to live attenuated virus, tight control of YFV-17D replication by the host is likely still required to attenuate virulence and prevent evasion of the immune response while adaptive immunity is being established. However, the mechanisms governing this tight balance between viral attenuation and immunogenicity are incompletely characterized. Although the role of type I and type II IFN signaling has been previously explored in mouse models (7, 12, 13), the role of type III IFN signaling in controlling YFV-17D infection is less well defined. We found that when YFV-17D actively replicates, type III IFN is critical for controlling even attenuated YFV and for maintaining proper host immune responses. Indeed, a significantly larger fraction of αβR^−/−^ λR^−/−^ mice than αβR^−/−^ mice died upon YFV-17D infection. This phenotype was associated with severe neuroinvasion and increased BBB permeability. Abrogation of type III IFN signaling also significantly impacted the frequencies of multiple immune cell lineages upon infection and impaired T-cell activation, suggesting a profound impact of type III IFN on sustaining an effective host immune response while YFV-17D is actively replicating. Finally, type III IFN also appeared to be important for regulating proinflammatory cytokine secretion. Taken together, results from our study support a model in which severe viral neuroinvasion in αβR^−/−^ λR^−/−^ mice could be caused by the convergence of three major elements: active viral replication, increased BBB permeability, and immune function dysregulation (also associated with severe proinflammatory cytokine secretion) (Fig. 8A). Our work highlights the critical immunoregulatory and neuroprotective functions of type III IFN signaling for controlling replicating live attenuated flavivirus *in vivo*, as well as for maintaining a suitable immunological environment for the induction of a potent adaptive immune response.

Previous studies provided evidence that both YFV infection and YFV-17D infection are rapidly cleared by a type I IFN-mediated innate immune response in mice (7, 12, 13). Indeed, depletion of type I IFN allows extensive viral replication, leading to death or T-cell-mediated clearance during YFV-Asibi or YFV-17D infection, respectively (12, 16). Although YFV-17D inhibits type I IFN signaling in infected cells (15), evidence suggests that type I IFN might have a role in the YFV-17D-induced immunogenicity process in human vaccinees. Analysis of the transcriptome of peripheral immune cells from human vaccinees showed that several genes involved in type I IFN pathways (STAT1) as well as interferon-stimulated genes (ISG15, MX1, and OAS1) are upregulated upon vaccination (41). This correlated with immunogenicity, suggesting a link between the type I IFN response and immunogenicity. However, the precise nature of this link remains to be understood. Although it has been reported that type II IFN signaling is critical for controlling YFV-17D infection in mouse models where type I IFN signaling is abrogated, it was unknown how type III IFN signaling contributes to antiviral defenses (12). Unlike the IFN-αβ receptor, which is expressed on almost all nucleated cells, expression of the type III IFN receptor is restricted mostly to epithelial surfaces and a small panel of other cell types (20). Previous studies have demonstrated the ability of type III IFN to mediate an antiviral response at multiple epithelial barriers, such as the respiratory tract (42, 43), the gastrointestinal tract (44, 45), or the BBB (22). Type III IFN signalling, in contrast to type I IFN signalling, usually induces a limited set of interferon-stimulated genes (46), thus promoting a more moderate immune response in contrast with the broad, inflammatory type I IFN-mediated response. Despite its redundancy with type I IFN signaling, the evolutionary conservation of the type III IFN system likely relies upon its specific ability to induce a localized and low-potency immune response. Indeed, unlike most internal tissues, epithelial barriers are constantly exposed to external stimuli and microbes. The properties of type III IFN thus appear particularly adapted to protect these barriers, as the induction of a constant, broad type I-based inflammatory response would be detrimental for the host. Our study data suggest that during YFV-17D infection, type I IFNs represent a frontline immune barrier that likely activates a broad antiviral response. If this barrier breaks, type II and III IFN systems then represent two functionally and spatially distinct rescue mechanisms whose combined action is critical to maintain YFV-17D attenuation (Fig. 8A).

Both type I and type III IFNs have been identified in the regulation of BBB permeability and the prevention of viral neuroinvasion during infection of mouse models with WNV (22, 30). In the absence of type I or type III IFN signaling, viral replication appears to be a fundamental element in inducing BBB permeability during WNV infection (22, 30). Thus, the role of type III IFN signaling in regulating BBB permeability could not be properly assessed in λR^−/−^ mice, in which YFV-17D poorly replicated. However, even when potent viral replication was induced, no changes in BBB permeability were observed in the brain of αβR^−/−^ mice despite a more elevated amount of viral RNA than in that of WT and λR^−/−^ mice. As YFV-17D induced the secretion of multiple proinflammatory cytokines upon infection of αβR^−/−^ mice, our data also suggest an absence of competing effects between a protective type I IFN response and BBB-damaging cytokines in αβR^−/−^ mice, in contrast to WNV infection (30). Viral RNA was also found in the brain of infected WT and λR^−/−^ mice, suggesting a natural ability for a small fraction of YFV-17D particles to pass the BBB without disrupting it during clearance of peripheral infection. The increased numbers of viral RNA copies in the brains of αβR^−/−^ mice could thus reflect a more elevated level of viremia in the periphery instead of sustained neuroinvasion. Consistently, no significant increase in viral replication was observed in the brains of WT, λR^−/−^, and αβR^−/−^ mice between days 3 and 5 postinfection. This observation correlated with a more prominent number of infiltrating T cells in the brain of αβR^−/−^ mice, suggesting further viral clearance which was reflected by their survival following infection as well. Disrupting type III IFN signaling in αβR^−/−^ mice increased BBB permeability upon infection, suggesting an important role for type III IFN signaling in maintaining BBB integrity during extensive viral replication. Hence, instead of tightening the BBB as observed during neurotropic infection by flavivirus species (which naturally invade the BBB) (22), our data suggest that type III IFN signaling instead preserves the integrity of the BBB at its steady-state level upon viscerotropic flavivirus infection. A hypothetical model would be that type III IFN signaling preserves BBB integrity against the action of proinflammatory cytokines produced upon viral replication in αβR^−/−^ mice. Abrogation of type III IFN signaling would then neutralize any protection of the BBB and render the BBB susceptible to the actions of proinflammatory cytokines. The stark increase in IFN-γ levels in circulation, previously characterized as damaging for BBB integrity during rabies virus infection (40) and observed in αβR^−/−^ λR^−/−^ mice, could significantly contribute to the disruption of the BBB (Fig. 8B). Viral replication and type III IFN signaling therefore appear to be two major regulators that influence BBB permeability during viscerotropic flavivirus infection.

Beyond regulation of BBB permeability, our data also suggest that immunomodulatory functions of IFN-λ might be important to limit immune evasion and, consequently, neuroinvasion. Most of the variations in immune cell proliferation observed in αβR^−/−^ λR^−/−^ mice were also observed in λR^−/−^ mice, demonstrating that these changes were dependent on type III IFN signaling but not on extensive viral replication and associated changes in the inflammatory milieu. As type III IFN-specific changes in cell lineage proliferation were not observed in αβR^−/−^ mice, our data suggest that specific immunological changes induced by the depletion of type III IFN do not mirror an effective adaptive immune response but rather are part of a potential immune dysregulation process that is detrimental only in αβR^−/−^ λR^−/−^ mice. Additional variations in cell lineage frequencies were also observed only in αβR^−/−^ λR^−/−^ mice, suggesting that the depletion of both type I and type III IFN can synergistically perturb immune cell proliferation. How these changes in immune cell frequencies in multiple tissues can impact the functionality of the immune response will need further characterization. The strong type III IFN-dependent increase in the levels of cDCs, pDCs, MN, and NK and NKT cells in the spleen of αβR^−/−^ λR^−/−^ mice could have a significant impact of the proinflammatory/anti-inflammatory cytokine balance, as these subsets are major producers of proinflammatory cytokines. Numbers of B cells were significantly reduced in the blood and liver of αβR^−/−^ λR^−/−^ mice, suggesting a potential effect on B-cell-mediated control of YFV-17D infection. The frequencies of pDCs, which are major producers of IFN-β and IFN-α *in vivo* (47), increased in the periphery of αβR^−/−^ mice but not αβR^−/−^ λR^−/−^ mice upon YFV-17D infection, suggesting a potential defect in the antiviral response of pDCs in αβR^−/−^ λR^−/−^ mice. Finally, the stronger decrease in the numbers of CD4^+^ T cells observed in αβR^−/−^ λR^−/−^ mice in comparison to αβR^−/−^ mice and the strong increase in the numbers of CD8^+^ T cells in the liver of αβR^−/−^ λR^−/−^ mice point toward a potential perturbation of T-cell proliferation and activation. Consistently, αβR^−/−^ λR^−/−^ mice exhibited defects in T-cell activation in comparison to αβR^−/−^ mice, suggesting that type III IFN signaling enhances T-cell activation during YFV-17D infection. In αβR^−/−^ λR^−/−^ mice, this defect could promote immune evasion and, ultimately, viral neuroinvasion. Such a hypothesis is also supported by the fact that αβR^−/−^ mice displayed a more prominent number of infiltrating T cells in their brain than αβR^−/−^ λR^−/−^ mice, hence highlighting that a potential defect in T-cell-mediated immunity and T-cell migration could create an environment enabling more-extensive viral replication in the brain and viral neuroinvasion (Fig. 8B). Taking the data together, interfering with the immunomodulatory functions of type III IFN signaling could be detrimental to the host at two levels. First, it could impair a proper adaptive immune response, promote immune evasion, and hamper T-cell migration toward the central nervous system (CNS) (Fig. 8B). Second, dysregulation of the immune balance could cause tissue damage and inflammation, as reflected by potential cytokine activity on the BBB (Fig. 8B).

As mentioned above, the elevated concentration of IFN-γ in the blood of αβR^−/−^ λR^−/−^ mice could be a major factor in the pathogenesis observed in these mice. IFN-γ is mainly produced by Th1 CD4^+^ T cells and CD8^+^ T cells, as well as by ILC, NK, and NKT cells (9). This cytokine, along with other Th1 cytokines, can enhance BBB permeability during WNV or rabies virus infection (30, 40), likely by inducing the internalization of tight junction proteins at epithelial barriers (48). Type III IFN signaling has been reported to regulate the Th1/Th2 balance toward a Th1 bias (49, 50). However, disruption of type III IFN signaling appeared to stimulate, not reverse, this bias. We observed a strong increase in the numbers of IFN-γ producer cells in αβR^−/−^ λR^−/−^ mice, such as spleen-resident NK and NKT cells or liver NKT and CD8^+^ T cells, upon infection. This increase could favor immune evasion through continued stimulation of Th1 differentiation and IFN-γ production which would in return inhibit a Th2 response and B-cell-mediated immunity. We hypothesize that the Th1 bias, strong increase in IFN-γ levels, and disruption of BBB maintenance in αβR^−/−^ λR^−/−^ mice could thus be sufficient to induce the breakdown of the BBB (Fig. 8B). A Th1 bias would also favor virus immune evasion of a Th2 and humoral response, enabling spread to other nonvisceral tissues such as the central nervous system (Fig. 8B). Further studies will be required to precisely define how type III IFN signaling affects the production of IFN-γ and the Th1/Th2 balance and how their potential dysregulation can favor neuroinvasion upon YFV-17D infection.

Despite the strong conservation of physiologic and metabolic processes, mice and humans likely harbor significant differences in their ability to mediate host immune responses to viral infection (51). During YFV-17D infection, this is likely reflected by the species-specific interaction with the type I IFN system. In mice, type I IFN rapidly counteracts YFV-17D infection and is not required for the induction of a T-cell-specific immune response (12). In humans, YFV-17D blocks type I-IFN signaling in infected cells (15), and transcriptomic data from human vaccinees suggest a link between type I IFN signaling and human-specific immunogenicity (41). Mice and humans harbor differences in the IFN-λ genes that they express. Although mice harbor two functional IFN-λ genes, humans harbor four (20). One of them, IFN-λ4, is absent in mice but strongly conserved in other mammals, suggesting a functional role. Consequently, the role of type III IFN in YFV-17D attenuation described in this study will have to be validated further in more relevant experimental models, such as permissive primate species. However, such validation could be challenging due to the complexity of genetic engineering in such species. Despite differences between the mouse and human immune responses, rare cases of neurological adverse effects following YFV-17D infection have been observed in rhesus macaques and human vaccinees (reported rate in the United States, 0.8/100,000) (2, 24–26). Although death is rare and most human vaccinees recover from YFV-17D-induced neurologic effects, more than half of the cases have exhibited meningitis or encephalitis directly attributed to neuroinvasion (2). Instead of being associated with viral mutations—as YFV-17D has been shown to be extremely stable genetically (52)—these neurological effects are thought to be associated with increased host specificity and an innate immunity defect (2). Indeed, rare cases of YFV-17D-associated viscerotropic disease (reported rate in the United States, 0.4/100,000) (2) have been associated with single nucleotide polymorphisms (SNPs) in genes coding for critical actors in the innate immune response such as oligoadenylate synthetase (53) or RANTES (54). Thus, identifying whether these patients harbor SNPs in genes coding for IFN-λ or IFN-λ receptor could provide an additional explanation for this phenomenon. Indeed, IFN-λ SNPs have been associated with differences in clinical outcome during hepatitis C virus infection (56). Moreover, treatment with IFN-λ could represent a valuable option to counterbalance neurotropic manifestations in human vaccinees developing neurologic symptoms.

In conclusion, our study results suggest that type III IFN signaling is a central regulator of a tight immune balance that preserves both host immune functions and the integrity of the central nervous system during YFV-17D infection. Type III IFN thus appears to be a key host factor in controlling YFV-17D attenuation and potentiating immunogenicity.

## MATERIALS AND METHODS

### Mice.

C57BL/6 mice were originally acquired from The Jackson Laboratory (Bar Harbor, ME). Mice deficient for type I IFN receptor (α/βR^−/−^ mice), type III IFN receptor (λR^−/−^ mice), and type I and type III IFN receptors (α/βR^−/−^ λR^−/−^ mice) on a C57BL/6 genetic background were kindly provided by Sergei Kotenko (Rutgers University) and generated as described previously (55). All mice were bred in the Laboratory Animal Resource (LAR) Center of Princeton University. All animal experiments were performed in accordance to a protocol (number 1930) reviewed and approved by the Institution Animal Care and Use Committee (IACUC) of Princeton University.

### Cells and antibodies.

Huh-7.5 cells (kindly provided by Charles Rice, Rockefeller University, NY, USA) were grown in Dulbecco’s modified Eagle’s medium (DMEM) supplemented with 10% heat-inactivated fetal bovine serum (FBS; Thermo Scientific, Waltham, MA) and 1% penicillin-streptomycin (Thermo Scientific, Waltham, MA). The following anti-mouse antibodies (Abs) were used: from Life Technologies, Invitrogen (Foster City, CA), CD45-phycoerythrin (PE)-Texas Red clone 30F11 (dilution 1/100); from Biolegends (San Diego, CA), CD3-PE-Cy7 clone 17A2 (dilution 1/100) and CD4-Alexa Fluor 700 clone V4 (dilution 1/100); from BD Biosciences (San Jose, CA, USA), CD8-V500 clone 53-6.7 (dilution 1/100), CD11c-allophycocyanin clone HL3 (dilution 1/50), and CD45RA-PE clone 14.8 (dilution 1/50); and from Thermo Scientific/eBiosciences (San Diego, CA), CD19-Pe-Cy5.5 clone 1D3 (dilution 1/100), CD161-eFluor450 clone PK136 (dilution 1/50), CD11b-allophycocyanin-eFluor 780 clone M1/70 (dilution 1/100), F4/80-PE clone BM8 (dilution 1/100), CD317-Alexa Fluor 488 clone eBio927 (dilution 1/50), CD161-peridinin chlorophyll protein (PerCP)-Cy5.5 clone PK136 (dilution 1/50), CD197/CCR7-eFluor450 clone 4B12 (dilution 1/50), CD27-allophycocyanin-eFluor780 clone LG.7F9 (dilution 1/50), CD28-PerCP-Cy5.5 clone 37.51 (dilution 1/50), CD127-Alexa Fluor 488 clone A7R34 (dilution 1/100), CD62L-allophycocyanin clone DREG-56 (dilution 1/50), CD279/PD1-allophycocyanin-eFluor 780 clone J43 (dilution 1/50), and CD44-fluorescein isothiocyanate (FITC) clone IM7 (dilution 1/100).

### Infectious clone constructs, cloning, and *in vitro* transcription.

A pACNR-YFV-17D low-copy-number backbone (kindly provided by Charles Rice, Rockefeller University, NY) was transformed and amplified using low-recombination-frequency NEB 5-alpha high-efficiency competent *E. coli* cells (New England Biolabs, Ipswich, MA). Transformed bacteria were incubated in LB–50 μg/ml ampicillin (Sigma-Aldrich, Darmstadt, Germany) overnight at 30°C under conditions of shaking at 205 rpm. Plasmid cDNA was purified using an E.Z.N.A. endonuclease-free Maxiprep kit (Omega, Norcross, GA), ethanol (EtOH) precipitated, and linearized using Afi-II restriction enzyme (New England Biolabs, Ipswich, MA). Following concentration of linearized DNA by ethanol precipitation, viral RNA was transcribed from 1 µg of linear template using a mMESSAGE mMACHINE SP6 kit (Ambion, Foster City, CA) according to the manufacturer’s instructions.

### Electroporation and production of viral stocks.

Single-cell suspensions of Huh-7.5 cells were washed twice with Opti-MEM Gluta-Max-1 reduced-serum media (Life Technologies, Inc., Invitrogen, Foster City, CA) and resuspended at a concentration of 1.5 × 10^7^ cells/ml in Opti-MEM. Two micrograms of viral RNA was mixed with 0.4 ml of cell suspension and immediately pulsed in a 2-mm-path-length cuvette using an ElectroSquare Porator ECM 830 system (BTX, Holliston, MA) (860 V, 99 µs, five pulses). Electroporated cells were incubated at room temperature for 10 min prior to being transferred into 25 ml (P150 culture dish) of media. To produce a large-scale stock of YFV-17D, 5.4 × 10^7^ Huh7.5 cells were electroporated with 18 µg of RNA. At 24 h postelectroporation, the medium was changed and replaced by low-serum-concentration DMEM (1% FBS). Virus was collected at 48 h and 72 h postelectroporation. At 72 h postelectroporation, virus was pooled and concentrated 40-fold to 100-fold using Millipore 10,000-molecular-weight-cutoff (MWCO) spin filter columns (Merck Millipore, Darmstadt, Germany) (3,000 × *g*, 20 min) on the last day of collection. Viral titers were then assessed using a PFU assay.

### Titration of viral stocks and YFV-17D *in vitro* infections.

To determine the viral titer of the YFV-17D stock, 2.5 × 10^5^ Huh7.5 cells were seeded per well in a 6-well plate at 24 h postinfection. Serial dilutions of the viral stock from 10^−3^ to 10^−12^ were performed, and 2 ml of each dilution was incubated with Huh7.5 cells for 6 h at 37°C. At 6 h postinfection, media were replaced by a fresh Methocel solution (DMEM, 10% FBS, 1% methylcellulose). At 4 days postinfection, cells were washed with phosphate-buffered saline (PBS), fixed with 100% ethanol for 25 min, and stained with 0.1% crystal violet. The PFU count for each dilution was then determined.

### Mouse infection and monitoring.

C57BL/6, αβR^−/−^, λR^−/−^, and αβR^−/−^ λR^−/−^ adult male and female adult mice (2 to 6 months of age) were infected through intravenous injection in the tail vein with YFV-17D (10^6^ or 10^7^ PFU) diluted in 200 µl of PBS. Clinical manifestations of disease were monitored daily, and signs of clinical disease progression were recorded through weighing, clinical scoring, and temperature measurements using a rectal probe. Overall appearance was assessed using a clinical scoring matrix assigned as follows: 0, posture normal, appearance with smooth, shiny fur; 1, posture hunched, appearance with ruffled fur, loss of muscle tone, loss of weight; 2, posture hunched, trembling, shaky, appearance with ruffled fur, loss of weight, rash; 3, posture severely hunched, appearance disheveled, significant (greater than or equal to 20%) body weight loss; 4, death.

### Histologic analysis of liver and spleen tissue.

Liver and brain tissues from noninfected or infected (day 5 postinfection) wild-type, αβR^−/−^, λR^−/−^, and αβR^−/−^ λR^−/−^ mice were harvested and incubated in 4% (wt/vol) paraformaldehyde (PFA) at 4°C for 48 h and subsequently transferred into 70% (vol/vol) ethanol (EtOH). Tissue processing, paraffin embedding, and hematoxylin and eosin or CD3 staining (to stain T-lymphocyte) were performed by the Histopathology Reference Lab (HRL) (Hercules, CA) using the standard procedure. All liver and brain sections were of sufficient area to permit accurate examination of tissue histopathological manifestations and lymphocyte infiltration. For each mouse model and experimental condition (noninfected or infected), tissue (liver and brain [or brain only for lymphocyte infiltration analysis]) was examined using 6 tissue sections from three biological replicates (3 animals). The histopathological manifestations and extensive lymphocyte infiltration observed in αβR^−/−^ λR^−/−^ mice were absent from all examined noninfected animals and limited in other infected mouse models. Pictures depicting histopathological manifestations and extensive lymphocyte infiltration are representative of three biological replicates.

### Organ collection and isolation of immune cells.

Whole blood (200 µl) was collected through submandibular bleeding and transferred into EDTA capillary collection tubes (Microvette 600 K3E; Sarstedt, Nümbrecht, Germany). Cells were separated from plasma through centrifugation, and red blood cells were lysed with 1× lysis buffer (BD Pharm Lyse; BD Biosciences, San Jose, CA) for 15 min at room temperature in the dark. Following lysis and quenching with 10% FBS–DMEM, blood cells were then washed twice with a 1% (wt/wt) FBS–PBS solution before staining. At the indicated endpoints, mice were euthanized via exsanguination under ketamine/xylazine anesthesia. To isolate immune cells from spleen and liver tissues, spleens and livers were collected following sacrifice and individually placed in 15 ml of serum-free DMEM. Spleens and livers were then transferred into 6- and 10-cm-diameter dishes, respectively, mechanically dissociated using a razor blade, and digested with 5 and 10 ml of digestive buffer, respectively (Sigma-Aldrich, Darmstadt, Germany; 40 mM HEPES, 2 mM CaCl_2_, 2 U/ml DNase I, HBSS [Hanks’ balanced salt solution; Life Technologies, Inc., Invitrogen, Foster City, CA]) (0.1% [wt/vol] collagenase) for 30 min at 37°C. Following quenching with 10% (vol/vol) FBS–DMEM media, splenocytes were strained through a 100-µm-pore-size cell strainer and washed with 10% (vol/vol) FBS–DMEM twice. Splenocytes were then centrifuged and lysed with 1× lysis buffer (BD Pharm Lyse; BD Biosciences, San Jose, CA) for 15 min at room temperature in the dark. Following quenching, liver cell suspensions were spun at low speed (300 rpm) for 5 min and supernatants containing the intrahepatic lymphocytes were transferred into a new 50-ml tube. For each 25 ml of collected supernatant, 15 ml of lymphocyte separation medium (Corning Life Sciences, Tewksbury, MA) was slowly added to the bottom of each conical tube, and samples were centrifuged at 2,000 rpm for 20 min with no brake. The interphase between the two phases was then collected and transferred to a 15-ml conical tube and centrifuged at 1,200 rpm for 5 min. Splenocytes and liver-derived lymphocytes were then washed twice with a 1% (vol/vol) FBS–PBS solution and viable (trypan blue-negative) cells counted prior to staining.

### RNA extraction from serum and tissues.

At the indicated time points, 200 µl of blood was collected through submandibular bleeding in a 1.5-ml collection tube. Serum was then separated from blood cells by centrifugation (10 min, 3,500 rpm). Viral RNA was isolated from mouse serum using a ZR viral RNA kit (Zymo, Irvine, CA) according to the manufacturer’s instructions. At the indicated endpoints, mice were euthanized via exsanguination under ketamine/xylazine anesthesia. Spleen, brain, liver, kidney, and thymus tissues were collected, placed into RNAlater (Ambion, Foster City, CA) solution, and maintained overnight at 4°C. The isolated tissues (20 to 30 mg) were then resuspended in buffer RLT–1% β-mercaptoethanol (Qiagen, Hilden, Germany), lysed using a TissueLyser (Qiagen, Hilden, Germany) (20 cycles/s for 2 min, 1 min wait, 20 cycles/s for 2 min), and centrifuged at high speed (10,000 rpm) for 10 min. Total RNA was then extracted from the resulting supernatant using an RNeasy minikit (Qiagen, Hilden, Germany) following the manufacturer’s instructions.

### YFV-17D single-step reverse transcription–quantitative real-time PCR.

Viral RNA was quantified using single-step reverse transcription (RT)-quantitative real-time PCR (SuperScript III Platinum One-Step qRT-PCR kit; Life Technologies, Inc., Invitrogen, Foster City, CA) with primers and TaqMan probes targeting a conserved region of the 5′ untranscribed region (UTR) of the YFV-17D genome. Single-step RT-quantitative PCR (RT-qPCR) was accomplished in a StepOnePlus real-time PCR system (Applied Biosystems, Thermo Scientific, Waltham, MA) using the following thermal cycling procedure: 52°C for 15 min, denaturation at 94°C for 2 min, and 40 cycles of denaturation at 94°C for 15 s, annealing at 55°C for 20 s, and elongation at 68°C for 20 s. A cDNA sequence coding for the 5′ UTR was *in vitro* transcribed and used as a standard for the absolute quantification of viral RNA. The primers used were as follows: YFV-17D sense 1 (GCTAATTGAGGTGCATTGGTCTGC), YFV-17D sense 2 (GCTAATTGAGGTGTATTGGTCTGC), YFV-17D antisense 1 (CTGCTAATCGCTCAACGAACG), and YFV-17D antisense 2 (CTGCTAATCGCTCAAAGAACG). The probe used was YFV-17D probe (6-carboxyfluorescein [FAM]-ATCGAGTTGCTAGGCAATAAACAC-black hole quencher [BHQ]).

### Antibody staining and flow cytometry analysis.

A total of 2 × 10^6^ to 4 × 10^6^ peripheral blood mononuclear cells (PBMCs), splenocytes, or liver-derived lymphocytes were isolated as described above and stained for 1 h at 4°C in the dark with the appropriate antibody cocktail. Following washing (1% [vol/vol] FBS–PBS), cells were fixed with fixation buffer (1% [vol/vol] FBS–4% PFA–PBS) for 30 min at 4°C in the dark. Flow cytometry analysis was performed using an LSRII flow cytometer (BD Biosciences, San Jose, CA). Flow cytometry data were analyzed using FlowJo software (Treestar, Ashland, OR). Mouse immune cell subsets were gated as follows: for leukocytes, CD45^+^; for T cells, CD45^+^ CD3^+^; for CD4^+^ T cells, CD45^+^ CD3^+^ CD4^+^ CD8^−^; for CD8^+^ T cells, CD45^+^ CD3^+^ CD4^−^ CD8^+^; for B cells, CD45^+^ CD3^−^ CD19^+^; for NK cells, CD45^+^ CD3^−^ CD161/NK1.1^+^; for NKT cells, CD45^+^ CD3^+^ CD161/NK1.1^+^; for conventional dendritic cells, CD45^+^ CD3^−^ CD19^−^ CD11c^+^; for plasmacytoid dendritic cells, CD45^+^ CD3^−^ CD19^−^ CD317^+^; for monocytes, CD45^+^ CD3^−^ CD19^−^ CD11b^+^ CD11c^−^ F4/80^−^; and for macrophages, CD45^+^ CD3^−^ CD19^−^ CD11b^+^ F4/80^+^. To characterize T-cell activation, CD45^+^ CD3^+^ CD4^+^ or CD8^+^ T cells were analyzed for their expression of CCR7, CD45RA, CD27, CD28, CD127, CD44, CD62L, and PD1. Flow cytometry fluorophore compensation for antibodies was performed using an AbC anti-mouse bead kit (Life Technologies, Inc., Invitrogen, Foster City, CA). Counting beads were added to each sample prior flow cytometry analysis (AccuCheck Counting Beads, Life Technologies, Inc., Invitrogen, Foster City, CA).

### Cytokine quantification.

Cytokines were quantified using a LEGENDplex multianalyte flow assay kit (BioLegend, San Diego, CA). Serum from wild-type, αβR^−/−^, λR^−/−^, and αβR^−/−^ λR^−/−^ mice was incubated for 2 h at room temperature with customized premixed beads and detection antibodies specific for a panel of 12 murine cytokines (IL-23, IL-12, IFN-γ, TNF-α, KC, MCP-1, IL-1β, IP-10, IL-6, IL-33, IFN-β, and GM-CSF). Samples were then incubated for 30 min with SA-PE (BioLegend) and washed, and resulting fluorescent signals were analyzed on a flow cytometer (LSRII; BD Biosciences) according to the manufacturer’s instructions. Analyte concentrations were determined and heat maps were produced using LEGENDplex software (v7.0; BioLegend, San Diego, CA).

### Evans blue assay.

C57BL/6, αβR^−/−^, λR^−/−^, and αβR^−/−^ λR^−/−^ mice were infected through intravenous injection in the tail with 10^7^ YFV-17D PFU. Five days postinfection, mice were injected with 200 µl of 0.5% Evans blue solution (Sigma-Aldrich, Darmstadt, Germany) diluted in sterile PBS through intravenous tail injection. Thirty minutes later, mice were injected with 200 µl of ketamine/xylazine cocktail via intraperitoneal injection. Following administration of anesthesia, mice were sacrificed and brain, liver, and kidney were collected, placed into a formamide solution (Ambion, Foster City, CA), and incubated for 48 h at 55°C prior weighing performed with a high-precision balance. Tissues were then disrupted using a TissueLyser (Qiagen, Hilden, Germany; 20 cycles/s for 2 min, 1 min wait, 20 cycles/s for 2 min), complemented with an extra 300 µl of formamide, and centrifuged at high speed (10,000 rpm) for 10 min. Supernatants (40 to 50 µl) were then cleaned from the remaining tissue residues by being mixed with 95% (vol/vol) EtOH at a 1:3 ratio prior centrifugation (2,000 rpm, 5 min). Supernatants were then transferred into a flat-bottom 96-well plate (Nunc, Thermo Scientific, Waltham, MA), and absorbance was read at 620 nm. The same procedure was performed on tissues from cohorts of noninfected (wild-type, αβR^−^/^−^, λR^−^/^−^, and αβR^−^/^−^ λR^−^/^−^) mice to establish the permeability baseline for each tested tissue and mouse strain.

### Statistical analysis.

GraphPad Prism software (v6.0; GraphPad Software, Inc., La Jolla, CA) was used for statistical analysis. Statistics were calculated using Student’s *t* test and/or two-way analysis of variance (ANOVA) when appropriate. Statistical analyses of cytokine concentration levels were independently validated using a Mann-Whitney *U* test (*, *P* < 0.05; **, *P* < 0.01; ***, *P* < 0.001; ****, *P* < 0.0001).
