# Supplementary material for: Type III Interferon-Mediated Signaling Is Critical for Controlling Live Attenuated Yellow Fever Virus Infection In Vivo
Source: mBio. 2017 Aug 15;8(4):e00819-17. doi: 10.1128/mBio.00819-17 (PMC5559630; doi:10.1128/mBio.00819-17)

**Figure S1.** **Body temperature variations during YFV-17D infection.** Changes in body temperature of WT (black), λR -/- (green), αβR -/- (blue) and αβR -/- λR -/- (red) mice following YFV-17D infection using 10^7^ p.f.u. Solid line represent the mean for each time point and group of animals (n=4-5).


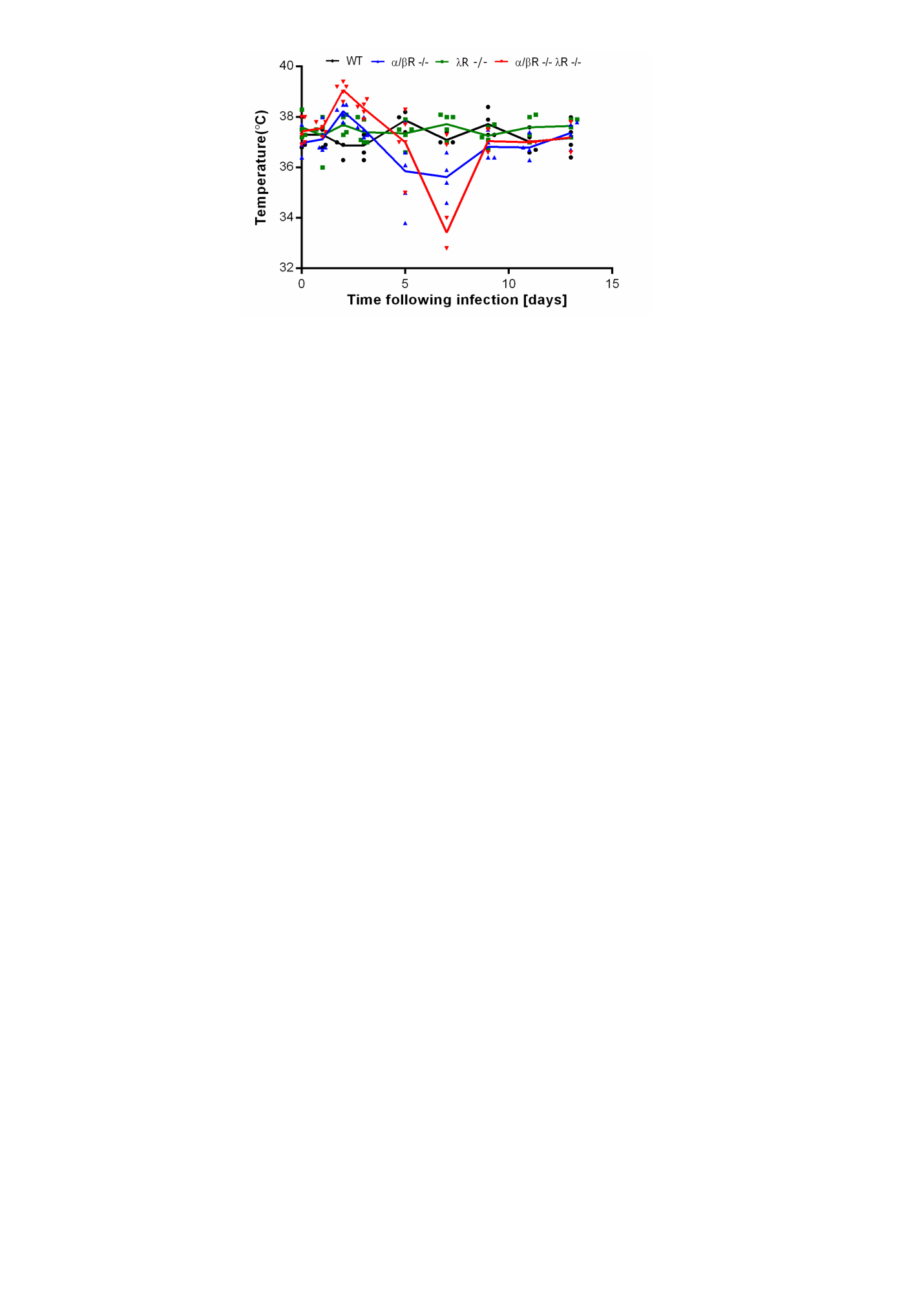

Supplement: FIG S1 [file mbo004173432sf1.docx]
