# Supplementary material for: Type III Interferon-Mediated Signaling Is Critical for Controlling Live Attenuated Yellow Fever Virus Infection In Vivo
Source: mBio. 2017 Aug 15;8(4):e00819-17. doi: 10.1128/mBio.00819-17 (PMC5559630; doi:10.1128/mBio.00819-17)
Supplement: FIG S2 [file mbo004173432sf2.docx]

**Figure S2. Type III IFN-mediated signaling regulate cell lineage proliferation in the blood and liver T-cell activation. A.** B cells and pDCs proliferation (total cell count per 10^5^ total murine CD45+) in the blood of non-infected (Mk, mock) or infected (17D) WT (black), λR -/- (green), αβR -/- (blue) and αβR -/- λR -/- (red) mice at day 5 following YFV-17D infection (10^7^ p.f.u.). Statistical analysis show the significant differences between non-infected (mock; Mk) versus infected (17D) conditions for each mouse model and cell lineage. (n=3 per group, **p*<0.05, ***p*<0.01, *****p*<0.0001). pDCs, plasmacytoid dendritic cells. **B-C.** Characterization of the activation phenotype of CD3+ CD4+ and CD3+ CD8+ T-cell populations in the liver of non-infected (Mk, mock) or infected (17D) WT (black), λR -/- (green), αβR -/- (blue) and αβR -/- λR -/- (red) mice at day 5 following YFV-17D infection (10^7^ p.f.u.). CD62L- CD44+ (**B**) and CD45RA- CD127- (**C**) populations were characterized among CD4+ and CD8+ T-cell population. Unless indicated otherwise, statistical analysis show the significant differences between non-infected (mock; Mk) versus infected (17D) conditions for each mouse model and cell lineage. (n=3 per group, **p*<0.05, ***p*<0.01, ****p*<0.001, *****p*<0.0001, ns = non-significant).
